# A simple design for microwave assisted digestion vessel with low reagent consumption suitable for food and environmental samples

**DOI:** 10.1038/srep37186

**Published:** 2016-11-17

**Authors:** Mehrdad Gholami, Shima Behkami, Sharifuddin Md. Zain, Sezgin Bakirdere

**Affiliations:** 1 Department of Chemistry, Marvdasht Branch, Islamic Azad University, P.O. Box 465, Marvdasht, Iran; 2 Department of Chemistry, University of Malaya, Kuala Lumpur 50603, Malaysia; 3 Department of Chemistry, Yıldız Technical University, 34220 İstanbul, Turkey

## Abstract

The objective of this work is to prepare a cost-effective, low reagent consumption and high performance polytetrafluoroethylene (PTFE) vessel that is capable to work in domestic microwave for digesting food and environmental samples. The designed vessel has a relatively thicker wall compared to that of commercial vessels. In this design, eight vessels are placed in an acrylonitrile butadiene styrene (ABS) holder to keep them safe and stable. This vessel needs only 2.0 mL of HNO_3_ and 1.0 mL H_2_O_2_ to digest 100 mg of biological sample. The performance of this design is then evaluated with an ICP-MS instrument in the analysis of the several NIST standard reference material of milk 1849a, rice flour 1568b, spinach leave 1570a and Peach Leaves 1547 in a domestic microwave oven with inverter technology. Outstanding agreement to (SRM) values are observed by using the suggested power to time microwave program, which simulates the reflux action occurring in this closed vessel. Taking into account the high cost of commercial microwave vessels and the volume of chemicals needed for various experiments (8–10 mL), this simple vessel is cost effective and suitable for digesting food and environmental samples.

Most trace analytical techniques need a homogeneous liquid sample to operate. Therefore, solid samples should be completely dissolved and digested prior to analysis. On the other hand, the digestion step is the Achilles’ heel of trace element analysis. If the digestion technique is too gentle, it will not break the sample matrix, therefore, the target analytes will not be released into the solution^1^. However, a method that is too harsh will not only destroy the sample matrix, but might also cause the loss of the analyte of interest. This is particularly the case with metals such as antimony, arsenic or tin^1^.

One of the techniques that is often used for digestion is ashing, which requires burning of the sample matrix until it becomes ash. The ash is usually soluble in an acid solution. This technique may also cause loss of analytes at high temperatures and yield poor results. Another difficulty is that some matrices do not easily turn into ash and may not even completely burn. Therefore, some of the target elements may not be taken into the solution^1^,^2^,^3^.

Fusion is another technique used for difficult matrices. This technique is very labor intensive and could be very expensive; furthermore, the high salt load and contamination problems due to the fluxing agent are the main disadvantages of this technique^1^,^2^,^3^.

Microwave digestion technique is an efficient, fast, and reproducible sample preparation method. In this method, the reaction timescale is dramatically reduced. Moreover, the potential for contamination decreases compared to open digestion techniques^3^,^4^,^5^,^6^,^7^,^8^,^9^,^10^,^11^,^12^. The first application of microwave in sample preparation was reported in 1975. In this study, a domestic microwave oven was employed to digest biological samples in an Erlenmeyer flask^13^. Soon after, scientists applied closed vessels to digest samples in higher temperatures and pressures^14^,^15^,^16^,^17^,^18^,^19^,^20^,^21^,^22^,^23^,^24^. All the closed vessels are armed with a safety system, which is either a safety membrane or a safety valve that opens instantly to control any increase in pressure by releasing some of the gases^1^. Therefore, volatile elements may be lost when gases are released from the closed vessels and this could be a reason for the lower recoveries in digestion^1^.

The amount of chemical reagent typically used for digestion of biological samples with commercial vessel/microwave in other studies is about 8–10 mL^25^,^26^; nevertheless, in this study, a maximum of 3.0 mL of chemical reagents are needed (2.0 mL of nitric acid and 1.0 mL of hydrogen peroxide).

Furthermore, the aim of this research was to design a simple PTFE vessel, which is safe to be used in a domestic microwave, as commercial ones might not be available for all due to its high cost. Finally, the performance of this design is evaluated with an ICP-MS instrument by analyzing the milk powder NIST 1849a, rice flour NIST 1568b, Peach Leaves NIST 1547 and Spinach Leaves NIST 1570a.

## Results

To evaluate the accuracy and applicability of the designed vessel in the proposed power to time program, the SRMs of milk powder, rice flour, peach and spinach leaves were digested and analyzed. The results found for these SRMs are compared with their corresponding certified values and reported in Tables (1, 2, 3, 4).

## Discussion

The results observed in Tables 1 and 2 shows excellent recoveries for most of the elements that exist in the milk powder and rice flour SRM samples. Nevertheless, the results shown in Tables 3 and 4 for the analysis of peach and spinach leaves SRMs, respectively revealed that the recoveries for Al, Cu, Zn, V, Mo, and Ni are not as good as the other elements. The lower recovery in this case might be contributed to the silicon content of peach and spinach leaves^6^; these elements are not completely released in microwave digestion as jelly silicon contents can keep part of the analytes while digestion is in progress^6^,^27^. This reduction in the recovery of elements, which was mentioned above, is directly related to the amount of silicon content of the leaves. The mean silicon content of peach NIST 1547 and spinach NIST 1570a leaves has been reported to be 979 and 1137 mg kg^−1^, respectively^6^. It is clear that lower recoveries for Al, Cu, Zn, V, Mo, and Ni observed in case of the peach and spinach leaves are caused by their higher content of silicon.

As explained, in order to prevent over pressure condition in the designed vessel, a domestic microwave oven was used, facilitated with inverter technology. In conventional microwave ovens, the power of microwave is always the maximum nominal power of the oven. Hence, for generating lower powers of radiation during the time of operation, the device was turned on and off successively so that the approximate required power of radiation can be transferred into the object. Nevertheless, in the microwave oven with inverter technology the exact required power of radiation can be applied continuously on the target.

The amount of chemical reagent typically used for digestion of biological samples is about 8–10 mL^25^,^26^; however, in this study, a maximum of 3.0 mL of chemical reagents (2.0 mL of nitric acid and 1.0 mL of hydrogen peroxide) were used to digest 0.10 g of milk powder, rice flour, spinach and peach leaves SRMs. The recommended procedure for the digestion of SRM 1849 – Infant/Adult Nutritional Formula is digestion of 1.00 g of this sample with 10.0 mL of HNO_3_ for the 75 mL MARSXpress vessel^28^. The results reported by the CEM corporation for this SRM are given in Table 5.

As it is clear the Agreement to (SRM) values (%) reported by recommended procedure of CEM corporation are relatively poorer than the results obtained in this study. This can be attributed to the amount of (SRM) sample, which is 1.0 g, and the amount of recommended HNO_3_, which is 10.0 mL. This amount of HNO_3_ and (SRM) sample can cause overpressure in the digestion vessel and activation of safety valve. Therefore, because of releasing over-pressurized gases, some of the target samples will be lost and this is the reason for poorer results reported by CEM corporation.

## Conclusions

A simple microwave digestion vessel design was introduced for digesting some food and environmental samples; the accuracy and applicability of this design was evaluated by the analysis of milk powder, rice flour, peach leaves, and spinach leaves SRMs. In this study a power to time microwave program, which simulates the reflux action occurring in this closed vessel were applied. Acceptable agreement to certified values have been observed for the analysis of milk powder and rice flour SRMs for most elements. However, it was observed that agreement to (SRM) values for some elements in peach and spinach leaves SRMs were not as good as milk powder and rice flour SRMs. It is concluded that this reduction in the recoveries as observed in some of the elements could be attributed to the silicon contents of these leaves. Moreover, the amount of chemicals used for the digestion in this vessel was reduced to 3.0 mL instead of 5 to 10 mL recommended by commercial vessels. By taking into account the price of commercial vessels and ultra-pure chemicals, these vessels can be considered as more cost effective and more environmentally friendly.

## Methods

The ICP-MS 7500ce (Agilent, USA) with an octopole reaction system (ORS) was used in this research. The Octopole Reaction System (ORS) is a Collision-reaction cell CRC containing an octopole ion guide in a stainless steel vessel which pressurized with a gas. Collision-reaction cell CRC is a technology to reduce or eliminate the effect of interferences coming from polyatomic species. This can be done by passing the ion beam (just before the quadrupole mass filter) through a cell that can be pressurized with a collision gas of either a reactive gas (e.g., H_2_, NH_3_, O_2_) or an inert collision gas (e.g. He). Argon gas that was used throughout the experiment is of spectral purity of (99.999%). Each day prior to the beginning of the experiment, the instrument was tuned with 1.0 μg L^−1^ (Agilent, USA) tuning solution containing Li, Co, Y, Ce and Tl in 2.0% (v/v) HNO_3_ and 0.50% (v/v) HCl to cover the entire mass range to assure proper sensitivity. The settings of the instrument are reported in Table 6.

A 1000 W (Panasonic, Japan) microwave oven NN-ST651M, 32 L with inverter and ∅340 mm turntable was used throughout the experiments. An (Elga Purelab Uhq II UK) system was used to produce ultra-pure water with resistivity more than 18 MΩ.cm.

**Figure 1:**
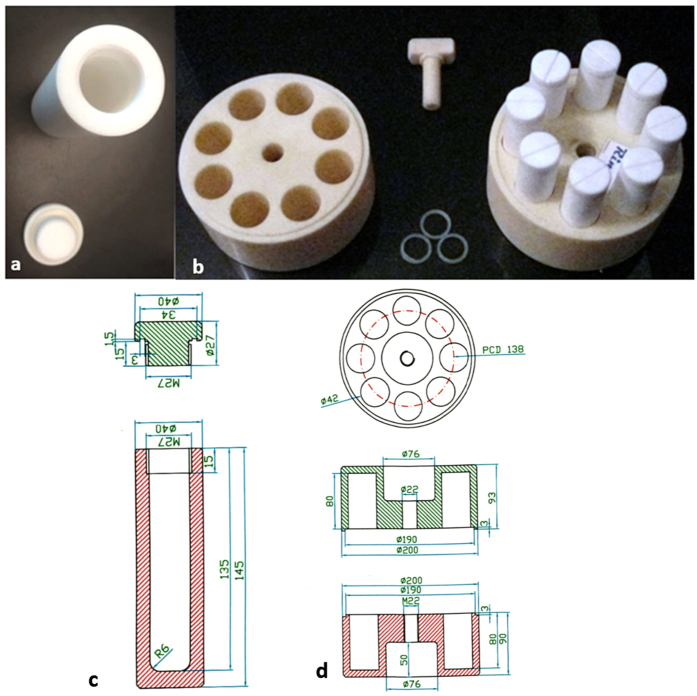
(**a**) Top view of a PTFE vessel, (**b**) PTFE vessels with their ABS casing, (**c**) vessel geometry, (**d**) (ABS) holder geometry.

A laboratory made polytetrafluoroethylene (PTFE) vessel was designed with a relatively thicker wall compared to commercial vessels while a silicone based polymer O-ring was used as a safety valve. The suggested vessel has been patented in Iran with patent no. 71522-1390/06/26, 2011 (Rima Instrument, Iran). In this design, eight vessels were placed onto an acrylonitrile butadiene styrene (ABS) holder to keep them safe and stable. The top view of the PTFE vessel is shown in Fig. 1(a); Fig. 1(b) represents the PTFE vessels with their ABS casing. The geometrical details of the vessel and their casing are shown in Fig. 1(c,d) respectively.

The reagents used for the analysis and digestion of samples are 60% ultrapure nitric acid and 31% ultrapure hydrogen peroxide (Merck, Germany). The standard reference materials were milk powder (Infant/Adult Nutritional formula obtained from NIST U.S department) 1849a, NIST rice flour 1568b, NIST peach leave SRM 1547 and NIST spinach leave SRM 1570a.

For the calibration plot, a multi element standard Agilent with concentration of 10 mg L^−1^ for Ag, Al, As, Ba, Be, Cd, Co, Cr, Cu, Mn, Mo, Ni, Pb, Sb, Se, Tl, V, Zn, Th and U, 1000 mg L^−1^ for Ca, Fe, K, Mg, Na and Sr were used. Concentrations of 50, 100, 300, and 1000 μg L^−1^ were used for five elements of Ca, Fe, K, Mg, Na and Sr, and concentrations of 0.5, 1, 3 and 10 μg L^−1^ for the rest of the elements. In Table 7 the limit of detection LOD, limit of quantitation LOQ, correlation coefficient, R^2^, and selected modes of gas are given. The LOD and LOQ values were obtained from 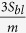 and 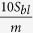 respectively, in these equations 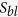 is the standard deviation of blank signal and *m* is the slope of calibration curve.

**Figure 2:**
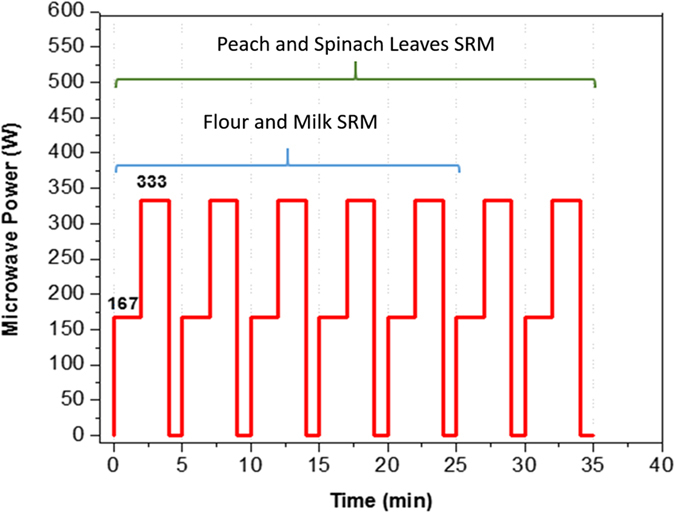
Power to time microwave program used for digestion of different SRMs.

0.10 g of each SRM sample was weighed and transferred into a PTFE vessel, then the vessels were put under the hood and 2.0 mL of ultra-pure 60% nitric acid plus 1.0 mL of ultra-pure 31% hydrogen peroxide were added into each vessel and the vessels were closed. A power to time microwave program was used for the digestion of standard reference materials. The details of power to time program used in this experiment are as shown in Fig. 2. This program was obtained by several trial and error varying the time to power program with constant amount of (SRM) samples (0.1 g) and digestion solution volume (3.0 mL) to gain maximum agreement with (SRM) values. In this program, the power of the microwave oven was increased stepwise, first to 167 W for 2 minutes and then it was increased to the maximum 333 W for 2 more minutes; afterwards the power was decreased to zero for one minute. This trend was repeated five times for milk powder and rice flour (SRMs) and seven times for peach and spinach leaves (SRMs). After digestion, the vessels were put under the hood to cool down. The contents were filtered through a 0.45 μm PTFE, if necessary (only peach and spinach leaves SRMs), and then transferred into a 50 ml polypropylene volumetric flask and diluted with ultrapure water to the marked level. The diluted samples were stored in polyethylene vials until the time of analysis by ICP-MS.

For cleaning, 3.0 mL of analytical grade nitric acid was poured into each vessel. After closing the vessels, a similar program with that used for digestion was applied to wash the vessels for 15 minutes.

This vessel armed with silicone O-ring to release any over pressure that may occur because of applying higher power of microwave radiation or longer time of applying radiation. However, in this power to time microwave program, it was tried to apply power and time of radiation to digest different standard reference materials in a way to prevent the overpressure condition. This was archived by several experimental cycles.

It has been reported that, when water is heated in a closed vessel microwave, the internal pressure is lower than that in closed vessels heated by conventional methods^4^,^26^. This is due to the vessel materials and the heating mechanism. Since the closed vessel microwave and their outer casing are microwave transparent, they remain relatively cool during the heating process. Therefore, the vessel’s wall becomes cooler and causes water molecules to remove from vapor phase^4^,^26^. Moreover, it was reported that simultaneous application of air flow outside the digestion vessel can improve the efficiency of digestion in the microwave oven^4^. Consequently, this enhancement in condensation rate causes a decrease in internal pressures at higher temperatures^4^,^26^. This is similar to a reflux action, which explains the better digestion. With the same conclusion, the cooling and heating period, shown in Fig. 2, simulates the reflux action and causes very good agreement to (SRM) values. These vessels armed with silicone O-ring to release any over pressure that may occur because of applying higher power of microwave radiation or longer time of applying radiation.

**Figure 3:**
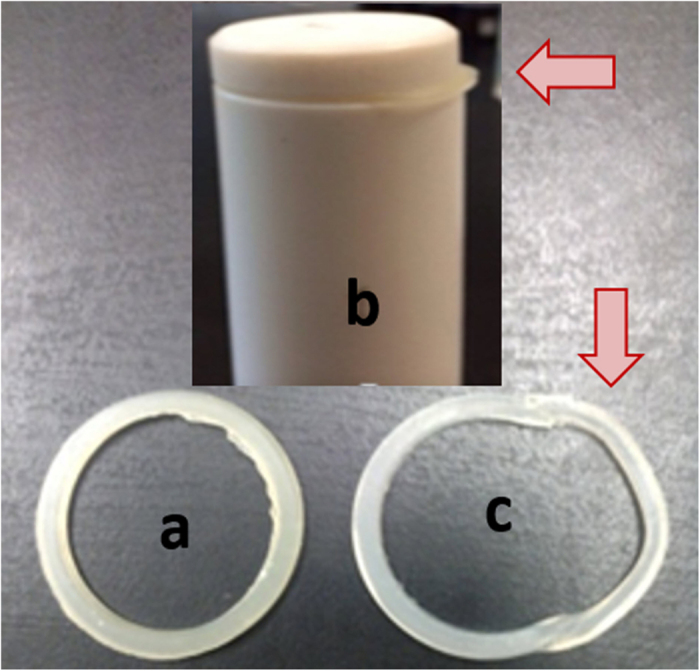
(**a**) Silicone rubber O-ring, (**b** and **c**) silicone rubber O-ring after deformation.

The PTFE vessels used in this research were equipped with a silicon-based polymer with high tensile and temperature resistance properties. In the case of enhancement of pressure at high temperature, this silicone rubber deforms to release the excess pressure. Figure 3(a) shows the silicone rubber O-ring before deformation and Fig. 3(b,c) show the silicone rubber after deformation, caused by high pressure and temperature.

It is necessary to mention here that although this vessel is armed with the safety system, the amount of sample (0.1 g) and the amount of reagents used for the digestion (3.0 mL) with the recommended time power program will not cause the activation of safety system of this vessel, therefore, better recoveries can be observed in this investigation.

## Additional Information

**How to cite this article**: Gholami, M. *et al.* A simple design for microwave assisted digestion vessel with low reagent consumption suitable for food and environmental samples. *Sci. Rep.* **6**, 37186; doi: 10.1038/srep37186 (2016).

**Publisher’s note:** Springer Nature remains neutral with regard to jurisdictional claims in published maps and institutional affiliations.

## Figures and Tables

**Table 1:** Comparison of the obtained values (mean ± uncertainty at the 95% level, 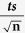) for milk powder SRM (1849a) with the reported certified values.

Elements	Concentration (mg.kg^−1^)			
	Certified value	Found value (n = 3)	RSD%	Agreement to (SRM) values (%)
Ca	5253 ± 51	5290 ± 177	1.35	100.7
Cr	1.072 ± 0.032	1.06 ± 0.02	0.94	98.9
Cu	19.78 ± 0.26	20.170 ± 0.005	0.01	101.1
Fe	175.6 ± 2.9	181.4 ± 10	2.28	103
K	9220 ± 110	9510 ± 224	0.95	103.1
Mg	1648 ± 36	1646 ± 32	0.78	99.9
Mn	49.59 ± 0.97	51.40 ± 0.71	0.56	103
Mo	1.707 ± 0.040	1.740 ± 0.020	0.57	102
Na	4265 ± 83	4379 ± 45	0.41	102
Se	0.812 ± 0.029	0.800 ± 0.020	1.25	98
Zn	151.0 ± 5.6	143.5 ± 4.8	1.35	95.1

**Table 2:** Comparison of the obtained values (mean ± uncertainty at the 95% level, 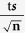 ) for Rice flour SRM (1568b) with the reported certified values.

Elements	Concentration (mg.kg^−1^)		RSD%	Agreement to (SRM) values (%)
	Certified value	Found value (n = 3)		
Ca	118.4 ± 3.1	119.1 ± 4.4	1.5	100.6
Cd	0.0224 ± 0.0013	0.0219 ± 0.0012	2.3	98.1
Cu	2.35 ± 0.16	2.34 ± 0.09	1.6	99.6
Fe	7.42 ± 0.44	7.50 ± 0.43	2.3	101.1
K	1282 ± 11	1299 ± 32	0.98	101.3
Mg	559 ± 10	553 ± 12	0.85	98.9
Mn	19.2 ± 1.8	19.8 ± 0.3	0.6	103.2
Mo	1.451 ± 0.048	1.441 ± 0.021	0.6	99.3
Na	6.74 ± 0.19	6.73 ± 0.16	0.98	99.8
Se	0.365 ± 0.029	0.368 ± 0.012	1.3	100.9
Zn	19.42 ± 0.26	18.72 ± 0.51	1.1	96.4

**Table 3:** Comparison of the obtained values (mean ± uncertainty at the 95% level, 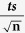 ) for peach leave SRM (1547) with the reported certified values.

Elements	Concentration (mg.kg^−1^)		RSD%	Agreement to (SRM) values (%)
	Certified value	Found value (n = 3)		
Al	249 ± 8	236 ± 4	1.56	94.8
As	0.060 ± 0.018	0.060 ± 0.008	11.6	100
Cd	0.026 ± 0.003	0.027 ± 0.001	3.7	103.8
Cu	3.7 ± 0.4	3.60 ± 0.06	1.47	97.3
Fe	218 ± 14	214 ± 5	2.1	98.2
Mn	98 ± 3	96 ± 1	1.25	98.1
Mo	0.060 ± 0.008	0.058 ± 0.002	3.44	96.7
Ni	0.69 ± 0.09	0.720 ± 0.009	11.1	104.3
Pb	0.87 ± 0.03	0.86 ± 0.03	3.48	98.8
Se	0.120 ± 0.009	0.120 ± 0.009	6.66	100
Sr	53 ± 4	54 ± 2	3.7	101.8
V	0.37 ± 0.03	0.35 ± 0.02	5.71	94.6
Zn	17.9 ± 0.4	16.80 ± 0.34	1.78	93.8

**Table 4:** Comparison of the obtained values (mean ± uncertainty at the 95% level, 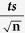 ) for spinach leave SRM (1570a) with the reported certified values.

Elements	Concentration (mg kg^−1^)		RSD%	Agreement to (SRM) values (%)
	Certified value	Found value (n = 3)		
Al	310 ± 15	280 ± 4	1.42	90.3
As	0.068 ± 0.012	0.065 ± 0.007	9.23	95.6
Cd	2.876 ± 0.058	2.690 ± 0.094	3.08	93.5
Cu	12.22 ± 0.86	11.88 ± 0.16	1.18	97.2
Mn	76.0 ± 1.2	74.8 ± 0.8	0.93	98.4
Ni	2.142 ± 0.058	1.970 ± 0.040	2.03	91.9
Se	0.1152 ± 0.0043	0.1158 ± 0.0080	6.04	100.5
Sr	55.54 ± 0.50	54.87 ± 1.80	3.28	98.7
V	0.568 ± 0.017	0.559 ± 0.040	7.15	98.4
Zn	82.3 ± 3.9	71.0 ± 1.2	1.54	86.3

**Table 5:** Comparison of the obtained values (mean ± uncertainty at the 95% level, 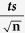 ) for SRM 1849 – Infant/Adult Nutritional Formula with the reported certified values reported by CEM corporation^28^.

Elements	Concentration (mg kg^−1^)		RSD%	Agreement to (SRM) values (%)
	Certified value	Found value (n = 3)		
Ca	4900 ± 130	4512 ± 207	1.85	90.08
Cu	20.29 ± 0.43	19.4 ± 0.42	2.04	95.43
Fe	177.1 ± 3.3	173.2 ± 9.1	2.15	97.82
Mg	1578 ± 69	1461 ± 71.8	1.97	92.59
Mn	51.00 ± 0.53	48.6 ± 2.5	2.02	95.22
Zn	152.3 ± 5.1	148.7 ± 7.2	1.96	97.65

**Table 6:** ICP-MS operating conditions.

Plasma (Ar) gas flow	15 L min^−1^
Carrier (Ar) gas flow	0.8–1.0 L min^−1^
Collision (He or H_2_) gas flow	4.5–5.0 L min^−1^
S/C temp	2 °C
Sampler and skimmer cons	Ni
Plasma Power	1500 W
Sample depth	6–8 min
Reflected power	<5.0 W

**Table 7:** ICP-MS analytical figures of merit.

Elements	LOD (μg. L^−1^)	LOQ (μg. L^−1^)	Gas Mode	R^2^
^27^Al	0.19	0.63	He	0.9996
^75^As	0.0091	0.0303	He	1.0000
^43^Ca	20.62	68.73	He	0.9992
^111^Cd	0.0035	0.0116	No Gas	0.9999
^52^Cr	0.012	0.040	He	1.0000
^65^Cu	0.013	0.043	He	1.0000
^56^Fe	0.23	0.77	He	1.0000
^39^K	3.41	11.37	He	1.0000
^24^Mg	0.16	0.53	He	1.0000
^55^Mn	0.064	0.213	He	1.0000
^95^Mo	0.016	0.053	He	1.0000
^23^Na	0.35	1.16	He	1.0000
^60^Ni	0.046	0.153	He	1.0000
^209^Pb	0.0092	0.0306	No Gas	0.9994
^78^Se	0.0029	0.0097	H_2_	1.0000
^88^Sr	0.041	0.1367	He	0.9998
^51^V	0.178	0.593	He	1.0000
^64^Zn	0.038	0.127	He	0.9975
